# Analysis of lymphocytic leukemia trends among gender, race, age, and regional groups in the U.S. between 1999-2022: a CDC-WONDER database study

**DOI:** 10.3389/fonc.2025.1555949

**Published:** 2025-05-29

**Authors:** Shannon Blee, Bob Weng, Taylor Billion, Ali Bin Abdul Jabbar, Abubakar Tauseef, Mohsin Mirza

**Affiliations:** ^1^ Department of Internal Medicine, Creighton University School of Medicine, Omaha, NE, United States; ^2^ Department of Internal Medicine, Creighton University, Omaha, NE, United States

**Keywords:** lymphoid leukemia, chronic lymphoid leukemia, acute lymphoid leukemia, oncology, disparities, trends, databases

## Abstract

**Background:**

Lymphocytic leukemia (LL) is a prominent group of hematological cancers afflicting both children and adults of all backgrounds and demographics. While treatment is improving, the confounding variables on mortality and prevalence within the patient population are poorly understood. This study utilizes the Center for Disease Control and Prevention (CDC)-WONDER database to further elucidate age-adjusted mortality rates (AAMRs) in the U.S. from 1999-2022.

**Methods:**

Mortality data was obtained from the CDC-WONDER database from 1999-2022. AAMRs and trends by gender, race, region, state, urban vs. rural, and age were analyzed using a Joinpoint analysis program to calculate average annual percentage change. Statistical significance was set at p ≤0.05.

**Results:**

Between 1999 and 2022, there was a decrease in overall mortality rates up until 2018, followed by an increase from 2018 to 2022. Men experienced higher age-adjusted mortality rates (AAMRs) than women, though women saw a greater decrease in mortality. Patients aged 85 and older had the highest crude mortality rates from 1999 to 2019. From 2019 to 2022, the White patients/White population had the highest AAMRs, while the American Indian/Alaska Native population experienced the largest increase in mortality between 2016 and 2022. Regionally, the Midwest and West consistently had higher AAMRs compared to other regions, with the Midwest having the highest AAMR and the smallest decline in mortality. From 1999 to 2019, Iowa saw the largest increase in AAMRs, while Kansas experienced the largest increase from 2019 to 2022. Rural areas consistently had higher AAMRs than urban areas throughout the period from 1999 to 2022, with both regions showing a decline in AAMRs starting in 2020.

**Conclusion:**

LL overall mortality decreased from 1999–2022 but varied significantly amongst demographic groups. The Midwest, rural, older, and non-Hispanic white populations experienced the largest mortality rates. Thus, policies and management plans should be developed accordingly to these biases in disparities.

## 1 Introduction

Lymphocytic leukemia (LL) is a group of hematological cancers involving the lymphocyte lineages, specifically the progenitors of B and T cells. It arises due to a series of somatic mutations in hematopoietic precursors which lead to clonal proliferation of lymphoblasts (1). Its clinical presentation entails predominantly unspecific features, including the constitutional B symptoms (e.g. fever, night sweats, and unexpected weight loss), anemia, arthralgia, lymphadenopathy, purpura, increased risk of infections, and hepatosplenomegaly (2–4). There are multiple different types of LL including acute (ALL), chronic (CLL), T-cell CLL, prolymphocytic leukemia, and hairy cell leukemia. ALL and CLL are the most common subtypes, with CLL composing 22-30% of leukemia, and ALL composing about 20% of leukemia. ALL mostly affects children and adolescents and can be further classified into either the T cell or B cell lineages (5, 6). Previous literature has also notably delineated a 20-fold increase in its prevalence in children with Down syndrome (7). Comparatively, CLL is largely prevalent among middle-aged and elderly adults, particularly males, and is currently the most common leukemia in that demographic in the Western World (8).

Since the 1950s, survival for patients with ALL has improved due to a combination of the development of better diagnostic criteria and treatment modalities (9). However, ALL incidence rates continue to rise with the factors contributing to this rise remaining largely unknown (10). On the other hand, CLL has experienced a remarkable decrease in incidence since 2013 (11). Patients demonstrated significant improvement in long-term survival following the introduction of multiple novel chemo immunotherapies brought to market since 2000 (11, 12). Despite these trends in incidence and mortality, the effects of confounding factors such as sex, race, age, and regional differences are poorly understood. Improvements in surveillance and documentation in the form of cancer registries and databases have enabled greater scrutiny into the nuances of cancer trends across extended periods of time.

Therefore, the purpose of this manuscript is to utilize the Center for Disease Control and Prevention (CDC)-WONDER database to further elucidate age-adjusted mortality rates (AAMRs) for LL in respect to sex, race, age, and regional variations in the U.S. between 1999 to 2022. Epidemiologically, many differences in ALL and CLL lie in the affected age groups and prognosis, however these are not the sole differences. Certainly, biological and genetic distinctions tailor management plans for each disease. When examining overarching sociodemographic trends, however, we believe the categorization of ALL and CLL into the broader scope of LL provides more comprehensive insight into cancer burden. While there has been substantial study of ALL and CLL, other LL subtypes and overall longitudinal LL trends should be further examined in the literature. With the (CDC)-WONDER database enabling the study of all LL subtypes, it is for these reasons our broader goal is to understand the overall general trends of LL, and thus the overall LL burden on the public and healthcare system, rather than individual subtypes of leukemia. Our findings of geographic and socioeconomic biases, healthcare officials and providers will better be able to establish and optimize policies and management plans to address existing disparities for LL patients.

## 2 Materials and methods

The CDC WONDER database was used to assess LL mortality in the United States from 1999 to 2022. The mortality data is derived from death certificates of U.S. residents. This database has been employed in numerous studies to analyze mortality trends for specific conditions, such as pneumonia and respiratory diseases. In this study, we utilized the International Classification of Diseases, 10th Revision, Clinical Modification (ICD-10) code C91 and tracked mortality trends in individuals aged 5 and older, up to 85 and beyond. It was chosen to do LL combined, rather than separate because this ICD-10 code encompasses eight different types of LL including, CLL, ALL, Hairy cell leukemia, subacute LL, prolymphocytic leukemia, adult T-cell leukemia, other lymphoid leukemia, and lymphoid leukemia unspecified. This ICD code allowed for investigating the general trends of LL from 1999-2022.

The CDC WONDER database was used to extract demographic data related to mortality, including gender, race/ethnicity, age, urban-rural, regions, and state. Racial/ethnicity groups were defined as non-Hispanic (NH) White, NH Black, NH American Indian/Alaskan Native, NH Asian/Pacific Islander, and Hispanic populations Age groups were defined as 5-14, 15-24, 25-39, 40-54, 55-69, 70 to 84, 85+ years of age. For urban-rural classifications, the National Center for Health Statistics Urban-Rural Classification Scheme was used to divide the population into urban and rural (13). The large central metro category is the most “urban” category, and the noncore category is the most “rural” category. The large central metro category contains counties in metropolitan statistical areas (MSAs) of more than one million, the large fringe category contains remaining counties of one million or more, counties in MSAs of 250,000-999,999 are medium metro, counties with MSAs under 250,000 are the small metro category, non-metropolitan counties were assigned to the noncore category (14). Regions were classified into Northeast, Midwest, South, and West according to the Census Bureau and Health and Human Services definitions.

Age adjusted morality (AAMR) for each demographic group and crude mortality were calculated. The calculation of the AAMR used the “2000 U.S. Standard.” The Joinpoint Regression Program (Joinpoint version 4.9.0.0 available from National Cancer Institute, Bethesda, Maryland) was used to determine trends in mortality from 1999-2022. Annual percentage change (APC) and average annual percent change (AAPC) with 95% confidence intervals (CIs) for the AAMRs were calculated for the line segments linking a Joinpoint using the Monte Carlo permutation test. APC and AAPCs were considered increasing or decreasing if the slope describing the change in mortality over the time interval was significantly different from zero using 2-tailed t test. Statistical significance was set at p ≤0.05. Asterisks were used to denotate significance.

## 3 Results

### 3.1 Overall

**Figure 1:**
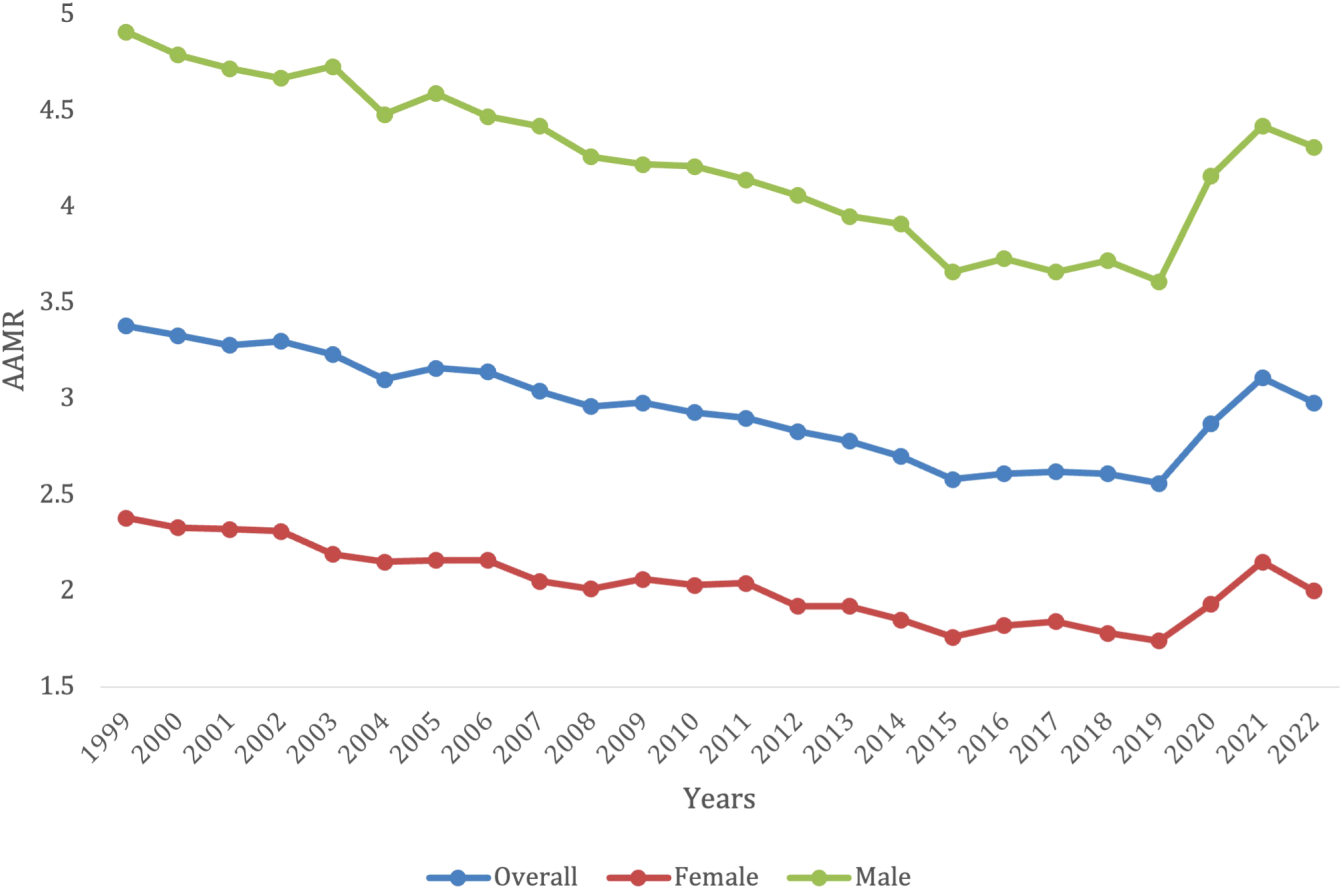
Trends in lymphoid leukemia age-adjusted mortality rates (AAMR) stratified by gender in the US, 1999-2022.

Overall, between 1999 to 2022, there were 221,009 deaths due to lymphoid leukemia in the United States. AAMR decreased from 3.38 (95% CI 3.31 3o 3.45) in 1999 to 2.56 (95% CI 2.51 to 2.61) in 2019 before increasing to 2.98 (95% CI 2.92 to 3.03) in 2022 (**Figure 1**; **Supplementary Table S1**). Throughout this period there was a significant decrease in overall lymphoid leukemia mortality with AAPC -0.42* (95% CI -0.63 to -0.23) (**Supplementary Figure S1**). There was a significant overall decrease in morality from 1999-2018 (APC, -1.49*, 95% CI -1.73 to -1.28), and a significant increase from 2018-2022 (APC, 4.81*, 95% CI 2.89 to 7.84) (**Supplementary Figure S1**).

### 3.2 Demographic differences

#### 3.2.1 Gender stratified

Overall, from 1999 to 2022, lymphoid leukemia caused 131,490 (59.5%) deaths in males and 89,519 (40.5%) in females in the United States. Both male and female mortality decreased until 2019, where they both began to increase again. For females, the AAMR decreased from 2.38 (95% CI 2.30 to 2.46) in 1999 to 1.74 (95% CI 1.69-1.80) in 2019, before increasing to 2.00 (95% CI 1.94 to 2.06) in 2022. For males, AAMR decreased from 4.91 in 1999 (95% CI 4.77-5.05) to 3.61 in 2019 (95% CI 3.51-3.70), before increasing to 4.31 in 2022 (95% CI 4.21-4.41) (**Supplementary Table S1**). Men had consistently higher AAMRs, when compared with females, yet females saw more of a decrease in mortality rate (AAPC -0.53* [95% CI -0.79 to -0.26] in females vs. -0.44* [95% CI -0.66 to -0.25] in males) (**Figure 1**). Males saw a significant decrease in mortality from 1999-2018 (APC -1.60*, 95% CI -1.85 to -1.37), but then it significantly increased to from 2018-2022 (APC 5.23*, 95% CI 3.20 to 8.43) from 2018-2022 (**Supplementary Figure S1**). Females saw a significant decrease in mortality from 1999 -2018 (APC, -1.56*, 95% CI -1.87 to -1.28), which then also significantly increased from 2018-2022 (APC 4.5*, 95% CI 2.12-8.43) (**Supplementary Figure S1**).

#### 3.2.2 Race stratified

**Figure 2:**
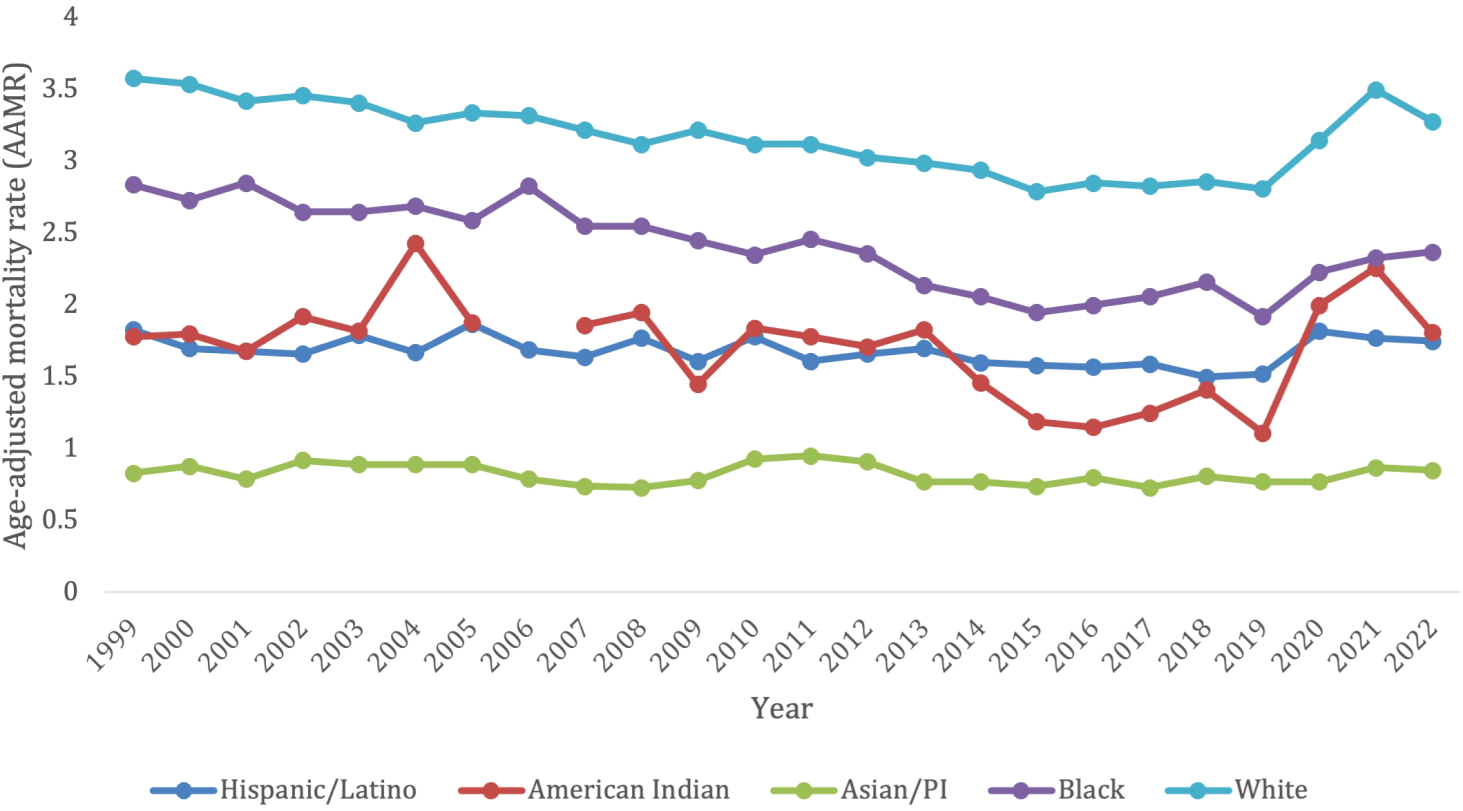
Trends in lymphoid leukemia age-adjusted mortality rates (AAMR) stratified by demographics in the US, 1999-2022.

Overall, the White patients/White population demographic had a total of 189,888 deaths from 1999-2022. The White patients/White population demographic had the highest AAMR over the years, with 3.58 (95% CI 3.50-3.67) in 1999 to 3.28 (95% CI 3.21-3.34) in 2022 (**Figure 2**). Notably, there was a significant spike in AAMR from 2019–2021 from 2.81 (95% CI 2.74-2.87) to 3.5 (95% CI 3.43-3.57), respectively, before decreasing in 2022 (**Supplementary Table S2**). This population did not have an overall significant decrease in morality from 1999-2022 (AAPC, -0.21, 95% CI -0.41 to 0.011). The White patients/White population demographic saw a significant decrease in morality from 1999-2018 (APC, -1.29*, 95% CI -1.52 to -1.07), but then had a significant increase in mortality from 2018-2022 (APC, 5.08*, 95% CI 3.23-7.82) (**Supplementary Figure S2**).

The Asian/Pacific Islander had 2,605 deaths due to lymphoid leukemia overall from 1999-2022. This population experienced an increase in AAMR from 0.83 (95% CI 0.62-1.09) in 1999 to 0.85 (95% CI 0.73-0.98) in 2022 (**Figure 2**). In contrast to all the other ethnic groups, the Asian/Pacific Islander population did not experience a surge in AAMR from 2019-2021 (**Supplementary Table S2**). Overall, this group did not have a significant change in mortality from 1999-2022 (AAPC, -0.33, 95% CI -0.98 to 0.44; APC -0.33, 95% CI -0.98 to 0.44) (**Supplementary Figure S2**).

Overall, from 1999–2022 the Hispanic/Latino population had 12,576 deaths due to lymphoid leukemia. They had a slight decrease in AAMR from 1.83 (95% CI 1.60-2.06) in 1999 to 1.75 (95% CI 1.62-1.88) in 2022. There was also a slight spike in AAMR from 2019–2021 from 1.52 (95% CI 1.39-1.64) to 1.77 (95% CI 1.63-1.90), respectively, before decreasing in 2022 (**Figure 2**; **Supplementary Table S2**). This population saw no significant change in overall mortality from 1999-2022 (AAPC 0.049, 95% CI -0.47 to 0.47). They saw a significant decrease in mortality from lymphoid leukemia from 1999-2018 (APC, -0.73*, 95% CI -1.80 to -0.27), but then saw a significant increase in mortality from 2018-2022 (APC, 3.81*, 95% CI 0.53 to -9.75) (**Supplementary Figure S2**).

Overall, from 1999-2022, the American Indian/Alaska native population saw a total of 707 deaths from lymphoid leukemia. This population saw a slight increase in AAMR from 1.78 (95% CI 1.04-2.85) in 1999 to 1.81 (95% CI 1.30-2.45) in 2022. There was a significant spike in AAMR from 2019–2021 from 1.11 (95% CI 0.71-1.65) to 2.26 (95% CI 1.65-3.01), respectively, before decreasing in 2022 (**Figure 2**; **Supplementary Table S2**). This population did not see any significant overall change in morality from 1999-2022 (AAPC, 0.51, 95% CI -0.37 to 2.01). They had a decrease in morality from 1999-2013 (APC -0.60, 95% CI -2.23 to 9.85), which significantly decelerated from 2013-2016 (APC, -14.42*, 95% CI -20.38 to -2.45), and then significantly accelerated from 2016-2022 (APC, 11.81*, 95% CI 6.99 to -24.64) (**Supplementary Figure S2**).

Finally, the Black patients/population had 16,550 overall deaths from lymphoid leukemia from 1999-2022. This population saw a decrease in AAMR from 2.84 (95% CI 2.61-3.06) in 1999 to 2.37 (95% CI 2.21-2.53) in 2022. Compared to other ethnic groups, there was not a pronounced spike in AAMR from 2019–2021 but AAMR has been gradually trending up from the nadir since 2019 at 1.92 (95% CI 1.78-2.07) (**Figure 2**; **Supplementary Table S2**). This was the only population to see a significant decrease in overall mortality from 1999-2022 (AAPC -0.83*, 95% CI -1.10 to -0.46). This population saw a decrease in morality from 1999-2011 (APC, -1.26, 95% CI -1.79 to 0.20), which then significantly decelerated from 2011-2015 (APC, -5.61*, 95% CI -9.04 to -2.66), and then significantly accelerated from 2015-2022 (APC, 2.78*, 95% CI 1.44-5.55) (**Supplementary Figure S2**).

### 3.3 Age group stratified

**Figure 3:**
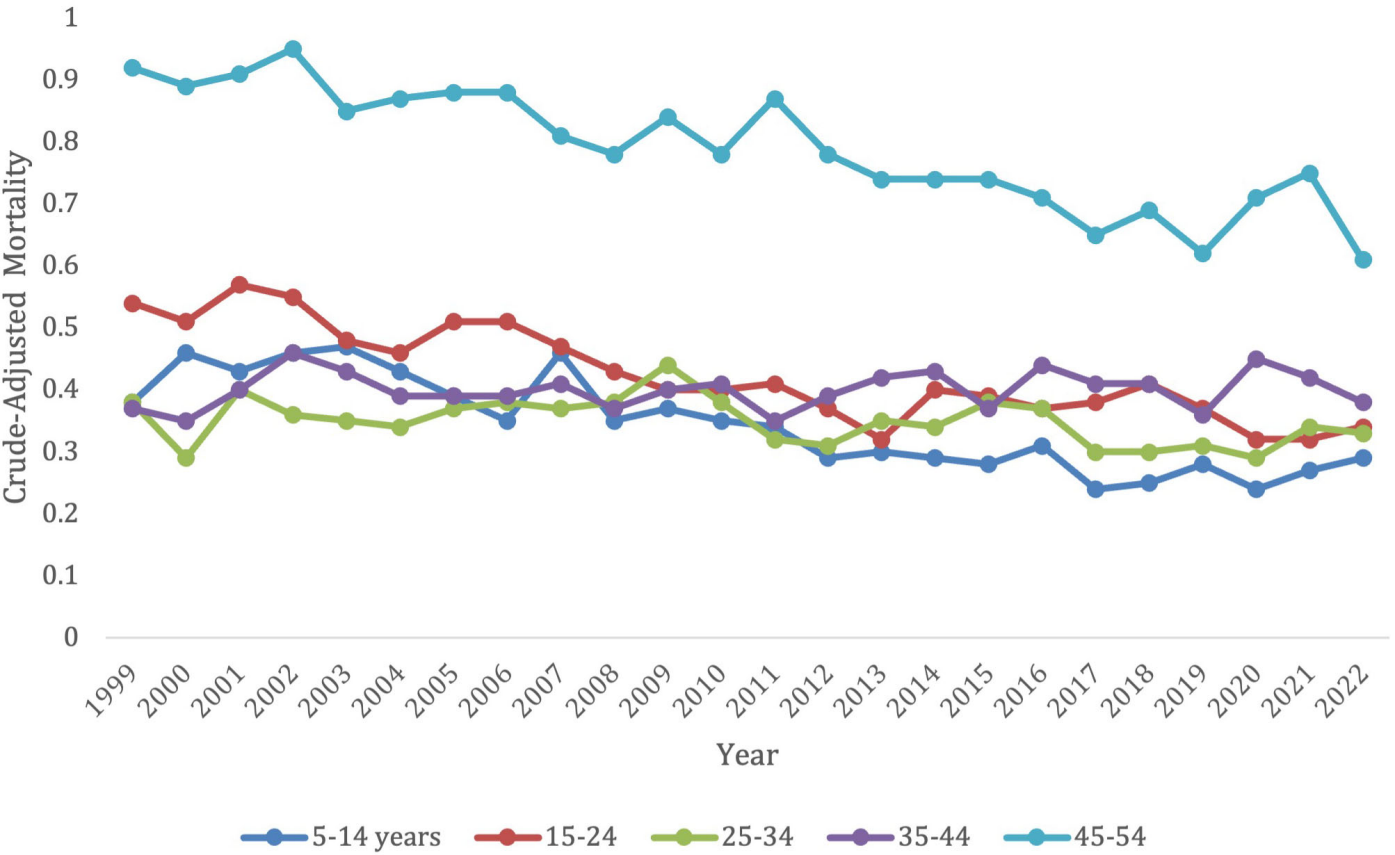
Trends in lymphoid leukemia age-adjusted mortality rates (AAMR) stratified by age in the US, 1999-2022 (ages 5-54).

Overall, 5–14 years old category had the lowest overall crude mortality rate, which remained steady from 0.38 (95% CI 0.32-0.44) in 1999 to 0.29 (95% CI 0.23-0.34) in 2022 without any significant changes from 2019-2021 (**Figure 3**; **Supplementary Table S3**). Those in the 5–14 age group, 15-24, and 45–54 age groups all had a significant decrease in overall mortality from 1999-2022 (AAPC, -2.89*, 95% CI -3.66 to -2.25; AAPC, -2.22*, 95% CI -2.80 to -1.72; AAPC, -1.54*, 95% CI -1.92 to -1.18, respectively) (**Supplementary Figure S3**). The 25-34- and 25-44-years old category both saw no significant change in mortality from 1999-2022 (AAPC, -0.65, 95% CI -1.41 to 0.04; AAPC, 0.16, 95% CI -0.38 to 0.68, respectively), and had no significant increase or decrease in morality during specific time periods.

The older age groups saw all had an original decrease in mortality, followed by an increase in later years. These older age groups, however, did all have a significant overall decrease in morality from 1999–2022 besides the 75–84-year-old category, which had no significant change during this time period (AAPC, -0.26, 95% CI -0.56 to 0.003). This 75–84 age group did though follow the trend of having a significant decrease in morality from 1999-2019 (APC, -1.25*, 95% CI -1.56 to -1.00) which increased from 2019-2022 (APC, 6.61*, 95% CI 2.90 to 12.51).

The 55–64 years old category had an overall crude mortality rate of 3.35 (95% CI 3.12-3.58) in 1999 to 2.06 (95% CI 1.92-2.19) in 2022, which remained steady without any significant changes from 2019-2021 (**Figure 3**; **Supplementary Table S3**). This age group had a significant overall decrease in morality from 1999-2022 (AAPC, -1.78*, 95% CI -2.21 to -1.21). They saw a decrease in morality from 1999-2010 (APC, -2.15, 95% CI -2.74 to 3.55), which significantly decelerated from 2010-2018 (APC, -4.00*, 95% CI -9.48 to -3.01), and then significantly accelerated again from 2018-2022 (APC, 3.91, 95% CI 0.003 to -11.61) (**Supplementary Figure S3**).

The 65–74 years old category had an overall crude mortality rate of 10.15 (95% CI 9.69-10.61) in 1999 to 6.59 (95% CI 6.31-6.86) in 2022, which remained steady without any significant changes from 2019-2021(**Figure 3**; **Supplementary Table S3**). This age group had a significant overall decrease in morality from 1999-2022 (AAPC, -1.47*, 95% CI -1.81 to -1.03) from 1999-2022. There was a decrease in morality from 1999-2012 (APC, -2.32, 95% CI -2.90 to 0.70), which significantly decreased from 2012-2017 (APC -5.45*, 95% CI -9.78 to -3.10), and then significantly accelerated from 2017-2022 (APC 4.99*, 95% CI 2.53-10.00) (**Supplementary Figure S3**).

The 85+ years old category had the highest overall crude mortality rate, which remained steady from 48.53 (95% CI 46.41-50.65) in 1999 to 50.45 (95% CI 48.73-52.16) in 2019, after which it experienced a substantial jump in crude mortality to its peak of 62.5 (95% CI 60.5-64.51) in 2021 before starting to decline to 60.47 (95% CI 58.58-62.36) in 2022 (**Figure 3**; **Supplementary Table S3**). This population saw a significant decrease in morality from 1999-2022 (AAPC, 0.76*, 95% CI 0.50-1.03). This age category had a significant decrease in morality from 1999-2015 (APC, -0.69*, 95% CI -1.19 to -0.27), which then significantly increased from 2015-2022 (APC, 4.16*, 95% CI 3.04-5.92) (**Supplementary Figure S3**).

### 3.4 Regional variation

#### 3.4.1 Census region-based differences

Overall, from 1999-2022, the Northeast had 41,493 deaths from lymphoid leukemia, the Midwest had 55,937 deaths, the South had 75,443 deaths, and the West had 48,136 deaths. Every census region had a decrease in lymphoid leukemia mortality from 1999-2018, and then every region saw an increase in mortality after 2020.

**Figure 4:**
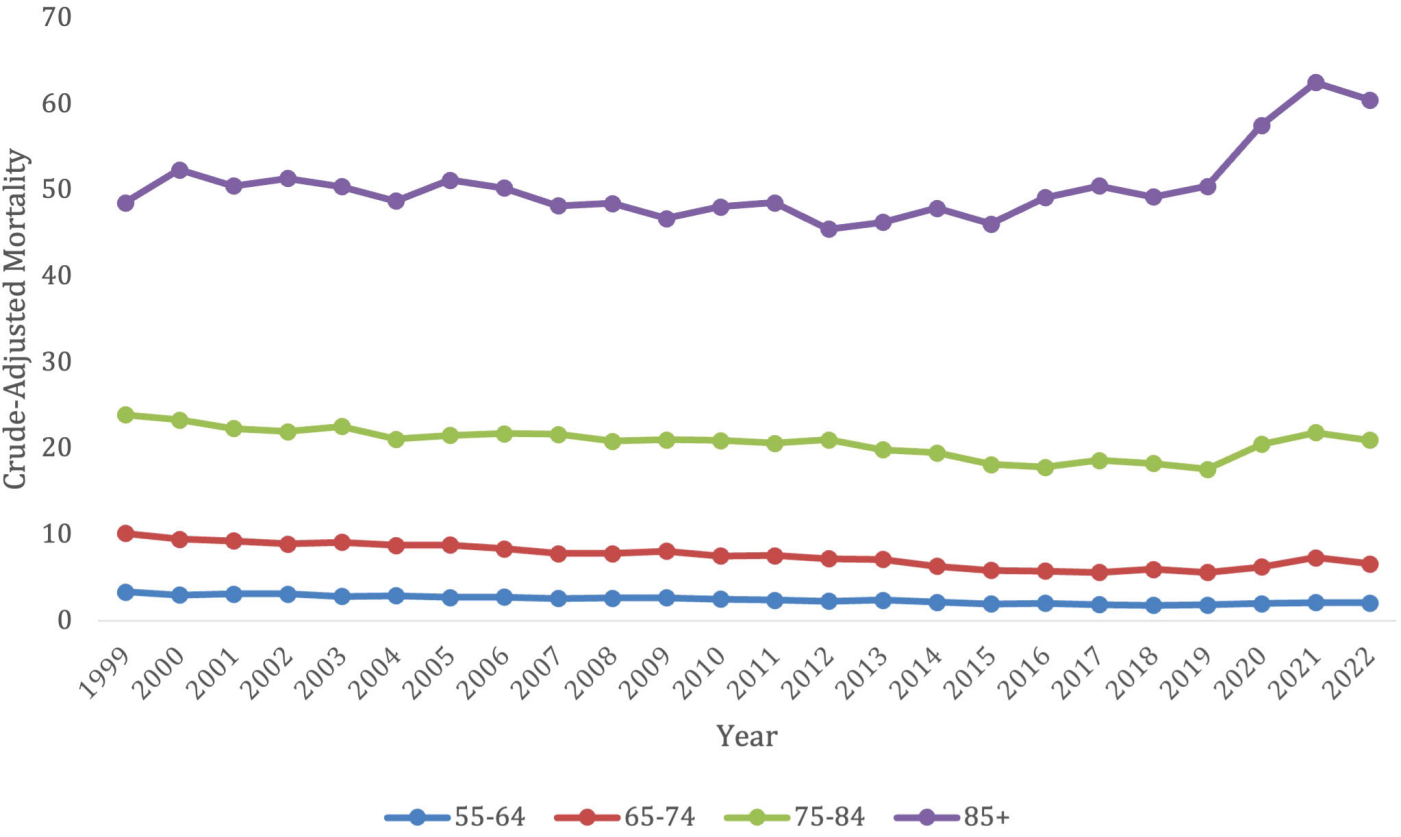
Trends in lymphoid leukemia age-adjusted mortality rates (AAMR) stratified by age in the US, 1999-2022 (ages 55-85+).

The Midwest and the West had consistently higher AAMRs compared to the Northeast and Southern regions, with the Midwest having the highest AAMR and smallest decline in mortality (AAPC in Midwest -0.33* [95% CI = -0.64 to -0.05] vs. in West -0.34* [95% CI = -0.64 to -0.05] vs. Northeast -0.57* [95% CI = -1.07 to -0.21] vs. West -0.34* [95% CI = -0.64 to -0.05]) throughout the time periods. All regions saw an increase in AAMR after 2020 (**Figure 4**; **Supplementary Table S4**).

Patients in the Midwest saw a significant decrease in overall mortality from lymphoid leukemia between 1999-2022 (AAPC -0.33, 95% CI -0.63 to -0.05), but the Midwest consistently had a higher AAMR than the other regions. The Midwest had an AAMR of 3.68 (95% CI 3.52 to 3.83) in 1999, which decreased to 3.43 (95% CI 3.3 to 3.56) in 2022. The Midwest saw a significant decrease in lymphoid leukemia associated mortality between 1999-2017, but then had a significant increase between 2017 and 2022, with APCs of -1.59 (95% CI -1.99 to -1.27) and 4.35 (95% CI 2.31 to 8.45) (**Supplementary Figure S4**).

Patients in the West also had a significant decrease in overall mortality from lymphoid leukemia between 1999-2022 (AAPC -0.34, 95% CI -0.64 to -0.05). The West had an AAMR of 3.39 in 1999 (95% CI 3.23 to 3.55), which decreased to 3.01 in 2022 (95% CI 2.89-3.13). The West also saw a significant decrease in lymphoid leukemia mortality between 1999–2018 and a significant increase between 2018-2022, with APCs -1.31 (95% CI-1.67 to -1.01) and 6.04 (95% CI 1.81 to 9.16) (**Supplementary Figure S4**).

Patients in the Northeast also had a significant decrease in overall mortality from lymphoid leukemia between 1999-2022 (AAPC -0.56, 95% CI -0.1.01 to -0.21), which was the greatest decrease amongst the regions. The Northeast had an AAMR of 3.32 in 1999 (95% CI 3.16 to 4.47), which decreased to 2.69 in 2022 (95% CI 2.56 to 2.81). The Northeast saw a significant decrease in mortality from 1999–2018 with APCs of -1.26 (95% CI -2.41 to -0.89). This region had no significant increase in mortality (**Supplementary Figure S4**).

Patients in the South had a significant decrease in overall mortality from lymphoid leukemia between 1999-2022 (AAPC -0.49, 95% CI -0.79 to -0.22). The South had an AAMR of 3.21 in 1999 (95% CI 3.09 to 3.33), which decreased to 2.86 in 2022 (95% CI 2.78 to 2.95). The South saw no significant increase or decrease in mortality during certain time periods (**Supplementary Figure S4**).

#### 3.4.2 State-level difference

**Figure 5:**
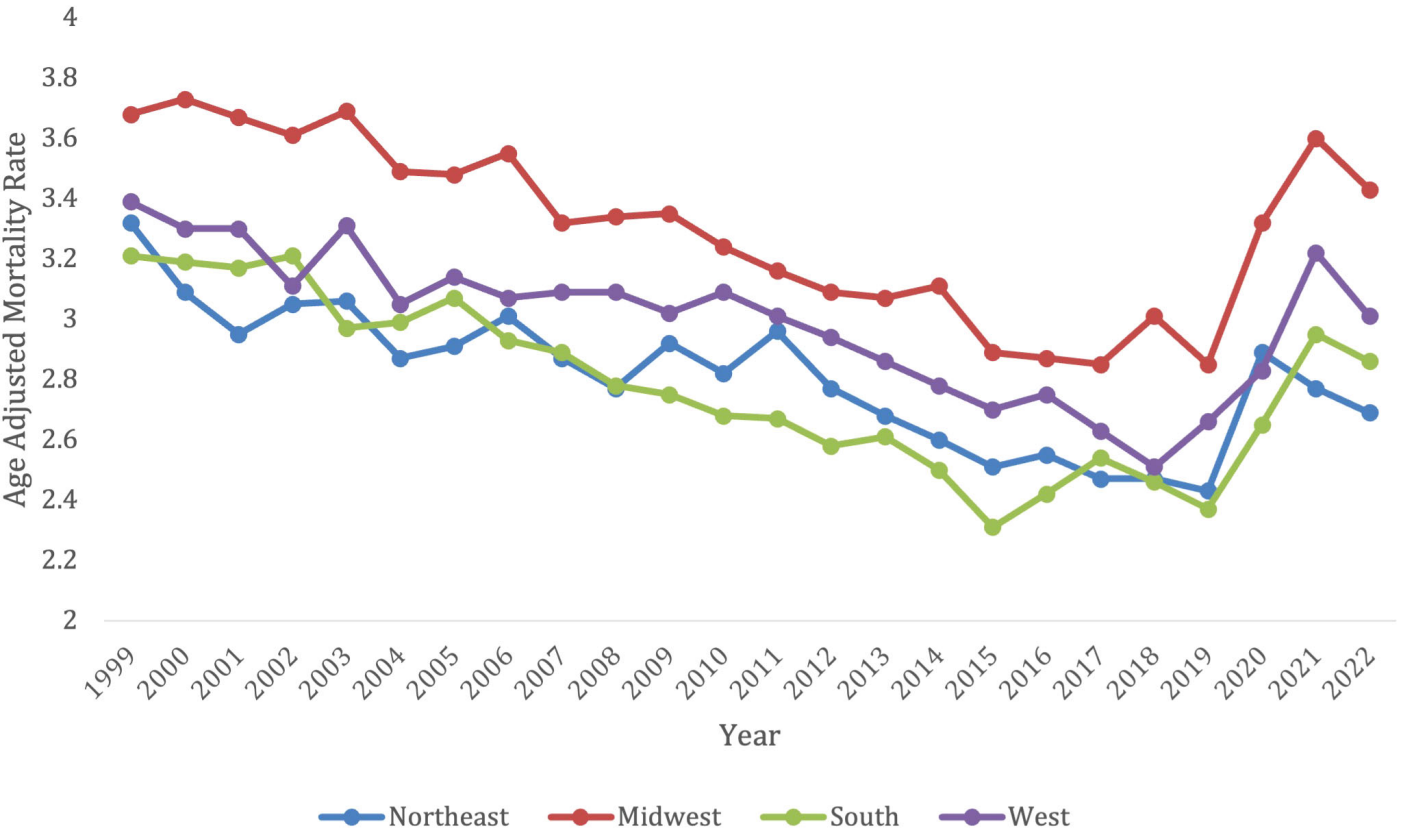
Trends in lymphoid leukemia age-adjusted mortality rates (AAMR) stratified by region in the US, 1999-2022.

From 1999-2019, Iowa had the largest increase in age-adjusted mortality rate related to lymphoid leukemia (0.24), followed by South Dakota (0.21) and North Dakota (0.03) (**Figure 5**). West Virginia saw the largest decrease in mortality (-1.55), followed by Kansas (-1.43) and Oregon (-1.28).

**Figure 6:**
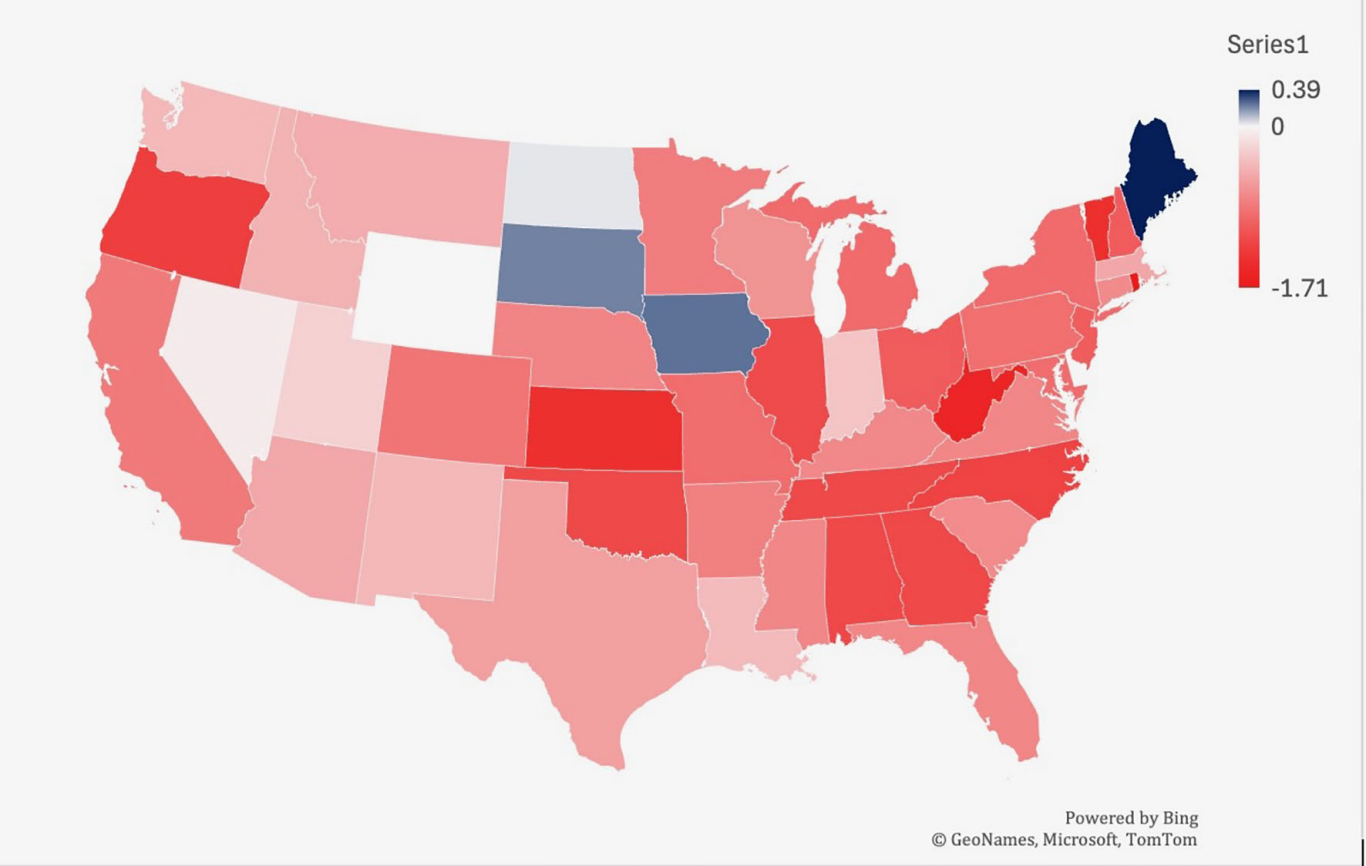
Trends in lymphoid leukemia age-adjusted mortality rates (AAMR) stratified by state in the US, 1999-2019. Lowest AAMR is indicated in red, and highest AAMR is indicated by blue.

From 2019-2022, Kansas had the largest increase in age-adjusted mortality at 1.47, followed by Rhode Island (1.46) and Delaware (1.28) (**Figure 6**). South Dakota saw the largest decrease in lymphoid leukemia mortality (-0.53), followed by Hawaii (-0.43) and Mississippi (-0.43) (**Figure 5**).

#### 3.4.3 Rural vs urban

**Figure 7:**
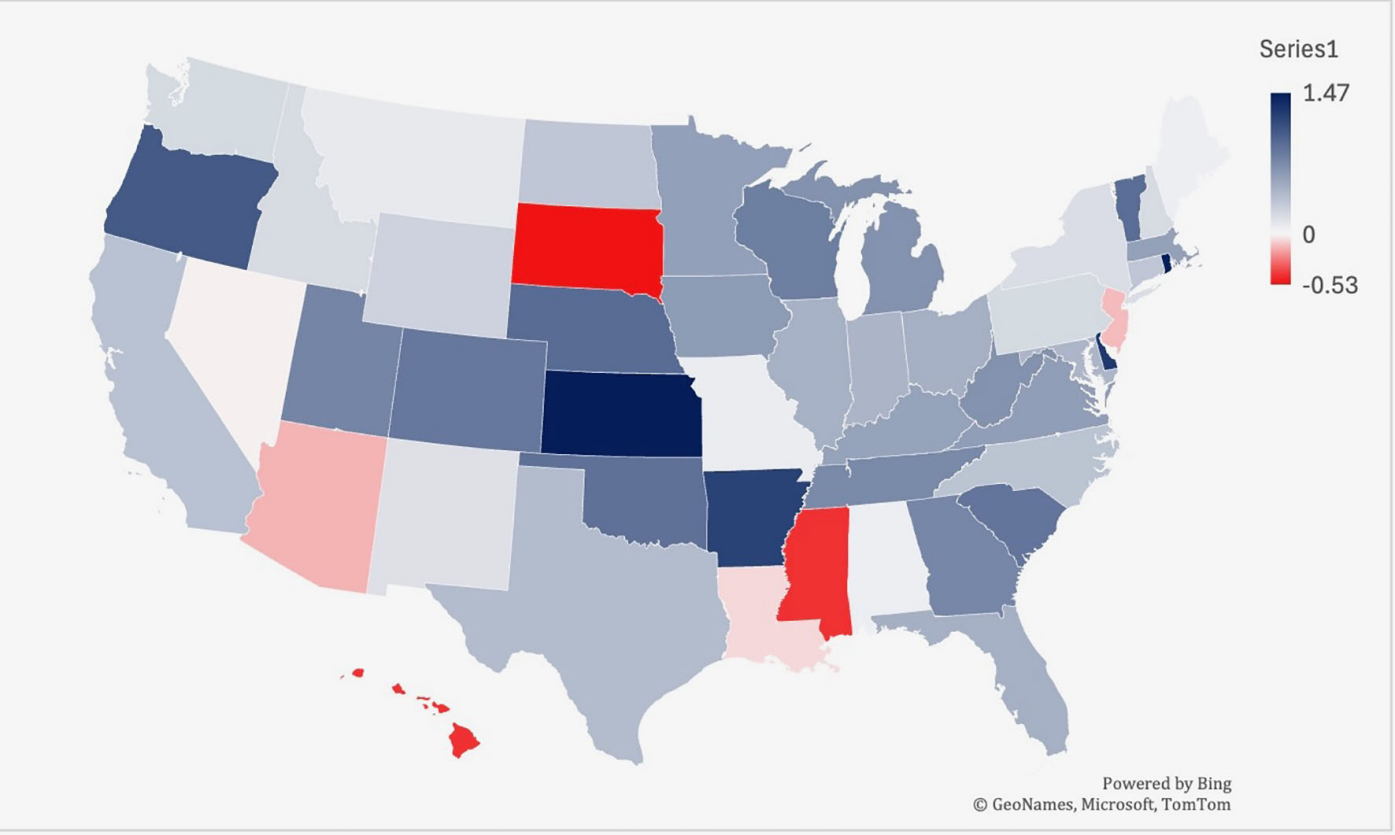
Trends in lymphoid leukemia age-adjusted mortality rates (AAMR) stratified by state in the US, 2019-2022. Lowest AAMR is indicated in red, and highest AAMR is indicated by blue.

Overall, from 1999-2020, there were 159,363 deaths in patients living in an urban setting, and 38,581 deaths in patients living in a rural setting. Yet, the rural setting had a higher AAMR for all years, when compared to an urban setting (**Figure 7**; **Supplementary Table S5**). The urban setting had a significant overall decrease in mortality from 1999-2020, compared to the rural which had an increase in mortality (AAPC -0.92* [95% CI -1.19 to -0.76] in urban vs. AAPC 0.64* [95% CI = 0.089 to 1.10] in rural).

When comparing populated regions, AAMRs were consistently highest in rural areas compared to urban regions. The rural population had an AAMR of 3.64 (95% CI 3.47 to 3.81) in 1999, which decreased to 3.3 (95% CI 3.15 to 3.44) in 2020. The rural regions saw an increase in mortality from 1999-2001 (APC 15.31*, 95% CI 8.59 to -17.86), which decelerated from 2001-2018 (APC -1.84*, 95% CI -2.77 to -1.48). The lymphoid leukemia morality then increased again from 2018-2020 (APC 8.59*, 95% CI 0.13-14.63) (**Supplementary Figure S5**).

In contrast, urban areas saw a decrease in mortality rates. The urban AAMR was 3.34 in 1999 (95% CI 3.27 to 3.42) and decreased to 2.8 in 2020 (95% CI 2.74 to 3.86). The mortality rates decreased from 1999-2018 (APC -1.48* 95% CI -1.70 to -1.30), but the significant increase from 2018-2020 (APC 4.49, 95% CI 0.68 to 6.27) (**Supplementary Figure S5**).

## 4 Discussion

There have been many advancements made in the treatment of lymphoid leukemia, which has led to a decrease in mortality over the years. However, in more recent years, there has been a steady increase in mortality, with many groups seeing an increase in mortality starting between 2018-2020. While there are a multitude of factors that may have contributed to this rise, one major contributor may have been the onset of the COVID-19 pandemic. The pandemic led to a large decrease in the number of cancer screenings and the amount of people accessing routine primary healthcare (15–17). Fewer patients were undergoing routine bloodwork, which is often one of the first places where the development of lymphoid leukemia can be detected (4). Additionally, as many primary care practices shifted to primarily virtual visits, this created a barrier for individuals without internet access or those struggling to navigate virtual platforms (18). These pandemic-related challenges may have contributed to delays in the diagnosis of lymphoid leukemia. For those who already had a diagnosis of LL, COVID-19 led to treatment delays and worsened clinical outcomes. One study investigating the length of time for EMS to make patient contact, found that it took significantly more time for EMS to make patient contact during COVID-19, which may have impacted life-threatening outcomes (19). In addition, multiple studies have shown that cancer patients had a harder time accessing treatments, delays in starting treatment and large scheduling delays, which may have contributed to increased disease burden and worsening mortality rates (20, 21). The increase in cancer mortality since the onset of COVID has been consistently seen across other cancers, such as breast, colorectal, esophageal and lung (22).

Regarding sex, males overall had consistently higher AAMRs, when compared to females, and females had a larger decrease in mortality. Both males and females saw a significant increase in mortality from 2018-2022, which may have been due to the onset of the pandemic during that time. It has been shown throughout literature that females consistently have had a decrease in incidence and better survival rates than men in patients with CLL (23), and that CLL tends to favor the male population. This is consistent for ALL as well, which has shown that males have had worse outcomes since the 1960s (24). Women may tend to have enhanced survival due to hormonal differences or decreased co-morbidities. Studies show that women also visit their primary care provider more frequently than men, which may lead to earlier detection and enhanced survival (25). However, the reason for these gender discrepancies remains largely unknown and further research is needed to understand it.

When comparing across races, the non-Hispanic White patients/White population had the highest AAMR over the years and had a large spike from 2019-2021. The American Indian/Alaskan Native population was the only other demographic to see a significant increase in mortality from 2019-2021. This may be explained by the disproportionate impact that the pandemic had on this community. Studies have shown that the American Indian community faces inadequate medical facilities, barriers to telemedicine, and underfunded resources (26, 27). All these factors may have led to worse outcomes for lymphoid leukemia patients throughout the pandemic. It will be important to monitor this trend for the next few years to determine if the mortality rate declines again. Interestingly, a previous study looking at SEER data from 1992–2007 found that the Black patients/population with CLL had worse survival than White patients/White population (28). While the results from this study show that non-Hispanic White population had the highest AAMR, the Black patients/Black population has been experiencing an increase in mortality since 2015. Therefore, it will be important to see if this continues to increase in the coming years.

Advanced age was associated with higher crude-mortality, with the 85+ years old category consistently having the highest crude morality rate compared to the other age groups. This may be because older populations are more likely to have increased co-morbidities that enhance their risk of mortality. In addition, many of the therapies that exist for the treatment of lymphoid leukemia, especially for ALL, are not as tolerable in the elderly population. Studies have shown that elderly patients are less likely to receive chemotherapy/TKIs/steroid treatment after being diagnosed with ALL, leading to increased mortality in those patients that receive no treatment (29). Many clinical trials in the past for CLL and ALL treatments fail to include the elderly population, which makes it challenging to determine the proper treatment for those with significant co-morbid conditions (30). Including more elderly patients in clinical trials to determine better treatment options may help to improve survival in this population.

**Figure 8:**
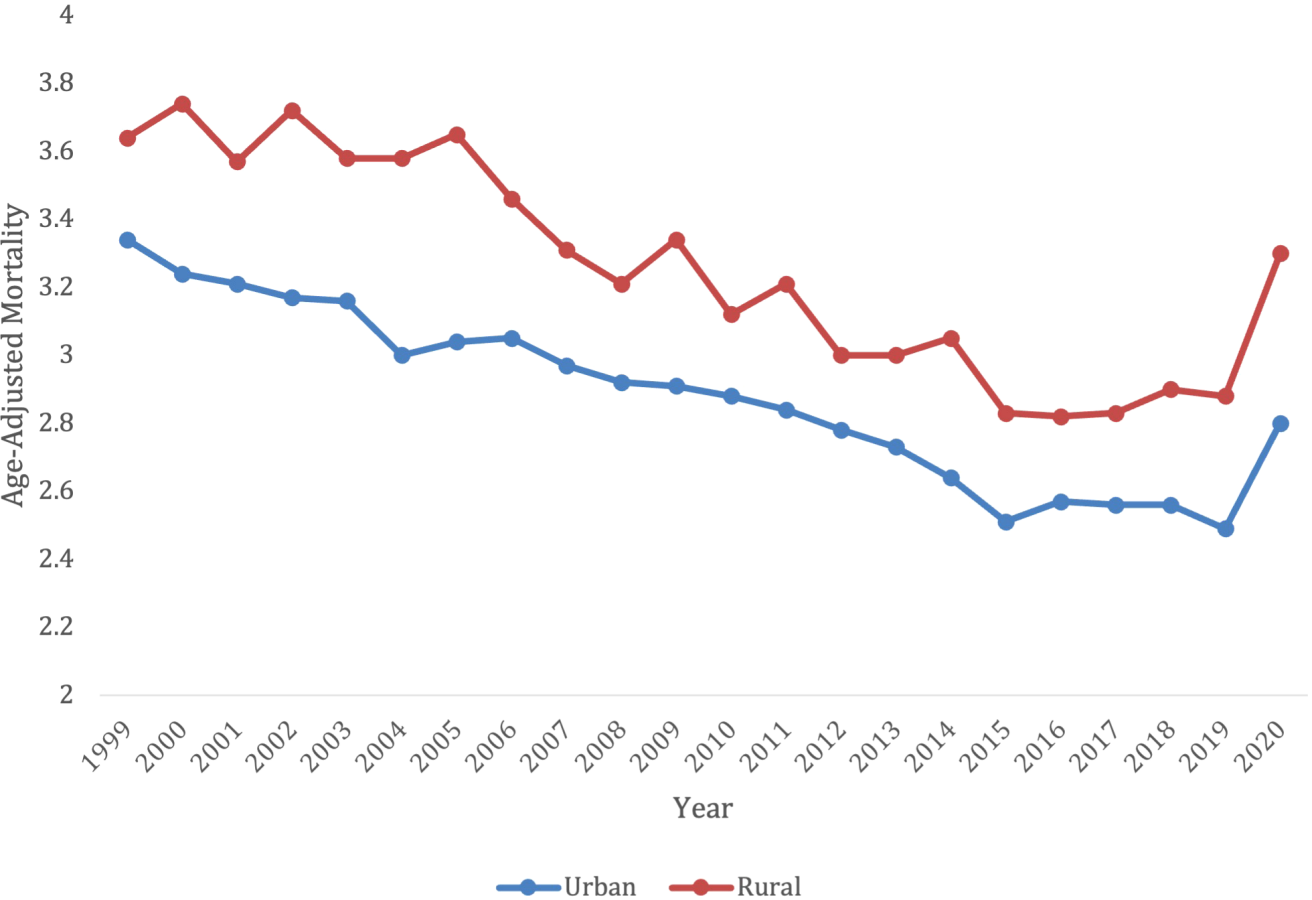
Trends in lymphoid leukemia age-adjusted mortality rates (AAMR) stratified by urban vs. rural in the US, 1999-2022.

In comparison to urban, the rural population had consistently higher AAMRs (**Figure 8**). Despite the propensity of rural communities consisting of largely older individuals, AAMRs remained higher in the rural population after adjusting for age discrepancies (31). Rather, the increase in AAMR is more likely due to extraneous factors, including the fact that rural populations tend to have longer travel distances to hospitals, increased co-morbidities, and a smaller likelihood of health insurance coverage (32). Studies have shown that rural populations have an increased disparities compared to urban populations for a multitude of cancers, not just lymphoid leukemia (33). All these factors may have contributed to the increase in mortality seen in the rural population. When comparing regions, the Midwest consistently had the highest AAMRs and had the smallest decline in mortality from 1999-2022. The Midwest is composed of a large proportion of rural and nonmetropolitan counties, with 64.4% of the rural population residing east of the Mississippi (34). States in the Midwest had the largest increases in mortality, with Iowa being the largest from 1999-2019. Interestingly, Kansas had one of the largest decreases from 1999–2019 but had the largest AAMR from 2019-2022. While it is challenging to elucidate exactly why Kansas saw this change, the increased mortality percentage of COVID-19 in the Midwestern states may have contributed (35).

Overall, the results from this study indicate that there are still large disparities that exist in mortality for lymphoid leukemia, with the largest disparities existing amongst the elderly and rural populations. Understanding these gaps can assist in working to alleviate these disparities and creating equitable care across differing demographic groups.

The CDC WONDER database has several limitations are important to note. This study examined LL trends and did not differentiate trends between CLL and ALL. Thus, general trends more accurately identify mutual factors affecting all LL as a whole rather than be applied to any specific subtype. Our study results are not appropriately able to be extrapolated for ALL or CLL alone, but only when considered together. Like any database study, reporting errors are plausible, involving categories such as death certificates, race, and residential regions. Moreover, cross-over analysis of the different parameters was not performed. For example, outcomes of different age groups among different races, sex, or state were not measured. In addition, the CDC WONDER database does not allow for a detailed analysis of demographic disparities, and therefore this study could not analysis the impact of specific socio-economic factors, such as income level, education, and health insurance coverage. However, this would be a good area for further research in the future. Several pockets of insufficient data were also present, such as the AAMR of Native Americans in 2006 or unavailable state data for Alaska, Delaware, District of Columbia, Hawaii, and Wyoming, which required extrapolation to make a presumed conclusion from the available data. Fourthly, demographic trends were limited by the breadth of the categories dictated in the study methodology. Urban-rural differentiation was done based on the National Center for Health Statistics Urban-Rural Classification Scheme which may not fully explain the nuances of different levels of urbanization or the existence of suburban populations and their relevance to the outcomes of LL.

## Conclusion

Overall, lymphoid leukemia mortality decreased between 1999-2022. However, there has been an increase in mortality in more recent years across many of the demographic groups, which may have stemmed from the COVID-19 pandemic and the limited access to healthcare that occurred during that time period. There also are differences in mortality associated with gender, race, regions and urban vs. rural communities. Therefore, it is imperative to find solutions to decrease the disparities in lymphoid leukemia in the United States, to enhance health equity amongst all demographic groups.

## Data Availability

The original contributions presented in the study are included in the article/**Supplementary Material**. Further inquiries can be directed to the corresponding author.
