# Supplementary material for: Analysis of lymphocytic leukemia trends among gender, race, age, and regional groups in the U.S. between 1999-2022: a CDC-WONDER database study
Source: Front Oncol. 2025 May 29;15:1555949. doi: 10.3389/fonc.2025.1555949 (PMC12158738; doi:10.3389/fonc.2025.1555949)
Supplement: Supplementary file 1 [file DataSheet1.docx]

Supplementary Material


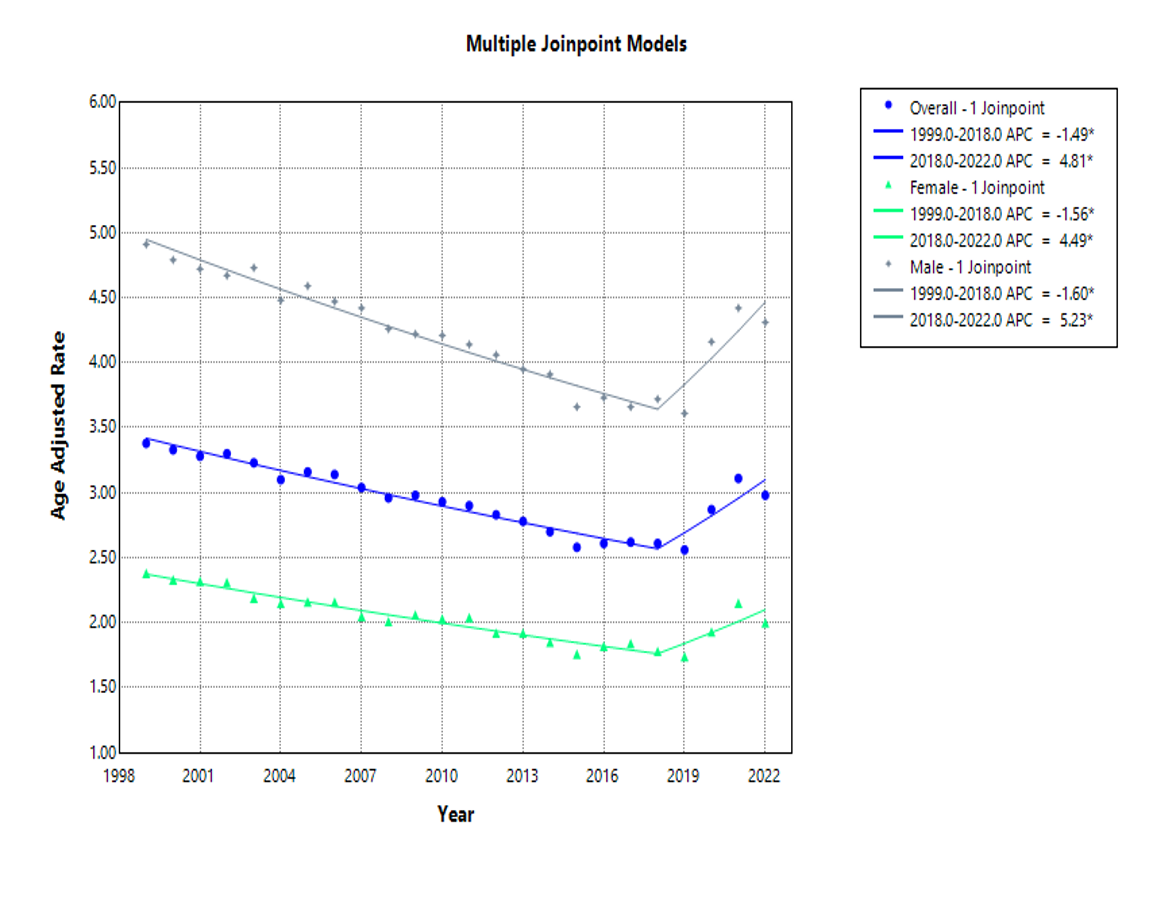


**Supplemental Figure 1**. Multiple Joint-Point Analysis stratified by gender in the US, 1999-2022.

| **Year** | **Overall** | **Female** | **Male** |
| --- | --- | --- | --- |
| 1999 | 3.38 | 2.38 | 4.91 |
| 2000 | 3.33 | 2.33 | 4.79 |
| 2001 | 3.28 | 2.32 | 4.72 |
| 2002 | 3.30 | 2.31 | 4.67 |
| 2003 | 3.23 | 2.19 | 4.73 |
| 2004 | 3.1 | 2.15 | 4.48 |
| 2005 | 3.16 | 2.16 | 4.59 |
| 2006 | 3.14 | 2.16 | 4.47 |
| 2007 | 3.04 | 2.05 | 4.42 |
| 2008 | 2.96 | 2.01 | 4.26 |
| 2009 | 2.98 | 2.06 | 4.22 |
| 2010 | 2.93 | 2.03 | 4.21 |
| 2011 | 2.90 | 2.04 | 4.14 |
| 2012 | 2.83 | 1.92 | 4.06 |
| 2013 | 2.78 | 1.92 | 3.95 |
| 2014 | 2.70 | 1.85 | 3.91 |
| 2015 | 2.58 | 1.76 | 3.66 |
| 2016 | 2.61 | 1.82 | 3.73 |
| 2017 | 2.62 | 1.84 | 3.66 |
| 2018 | 2.61 | 1.78 | 3.72 |
| 2019 | 2.56 | 1.74 | 3.61 |
| 2020 | 2.87 | 1.93 | 4.16 |
| 2021 | 3.11 | 2.15 | 4.42 |
| 2022 | 2.98 | 2.00 | 4.31 |

**Supplemental Table 1**. Age- Adjusted morality for overall and gender in the US from 1999-2022.


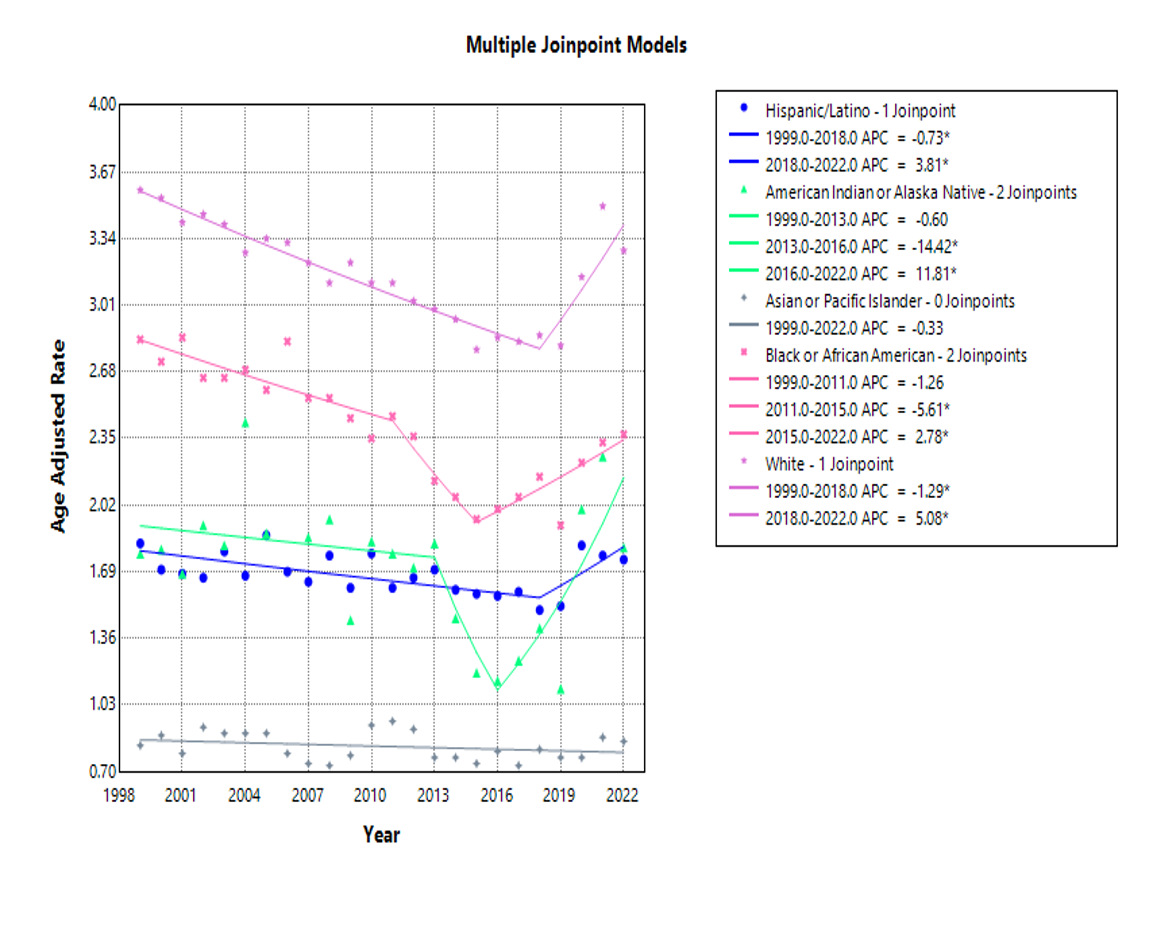


**Supplemental Figure 2**. Multiple Joint-Point Analysis stratified by race in the US, 1999-2022

| **Year** | **Hispanic/Latino** | **American Indian** | **Asian/PI** | **Black** | **White** |
| --- | --- | --- | --- | --- | --- |
| 1999 | 1.83 | 1.78 | 0.83 | 2.84 | 3.58 |
| 2000 | 1.70 | 1.8 | 0.88 | 2.73 | 3.54 |
| 2001 | 1.68 | 1.68 | 0.79 | 2.85 | 3.42 |
| 2002 | 1.66 | 1.92 | 0.92 | 2.65 | 3.46 |
| 2003 | 1.79 | 1.82 | 0.89 | 2.65 | 3.41 |
| 2004 | 1.67 | 2.43 | 0.89 | 2.69 | 3.27 |
| 2005 | 1.87 | 1.88 | 0.89 | 2.59 | 3.34 |
| 2006 | 1.69 | - | 0.79 | 2.83 | 3.32 |
| 2007 | 1.64 | 1.86 | 0.74 | 2.55 | 3.22 |
| 2008 | 1.77 | 1.95 | 0.73 | 2.55 | 3.12 |
| 2009 | 1.61 | 1.45 | 0.78 | 2.45 | 3.22 |
| 2010 | 1.78 | 1.84 | 0.93 | 2.35 | 3.12 |
| 2011 | 1.61 | 1.78 | 0.95 | 2.46 | 3.12 |
| 2012 | 1.66 | 1.71 | 0.91 | 2.36 | 3.03 |
| 2013 | 1.70 | 1.83 | 0.77 | 2.14 | 2.99 |
| 2014 | 1.60 | 1.46 | 0.77 | 2.06 | 2.94 |
| 2015 | 1.58 | 1.19 | 0.74 | 1.95 | 2.79 |
| 2016 | 1.57 | 1.15 | 0.80 | 2.00 | 2.85 |
| 2017 | 1.59 | 1.25 | 0.73 | 2.06 | 2.83 |
| 2018 | 1.50 | 1.41 | 0.81 | 2.16 | 2.86 |
| 2019 | 1.52 | 1.11 | 0.77 | 1.92 | 2.81 |
| 2020 | 1.82 | 2.00 | 0.77 | 2.23 | 3.15 |
| 2021 | 1.77 | 2.26 | 0.87 | 2.33 | 3.5 |
| 2022 | 1.75 | 1.81 | 0.85 | 2.37 | 3.28 |

**Supplemental Table 2**. Age-adjusted morality for race in the US from 1999-2022.


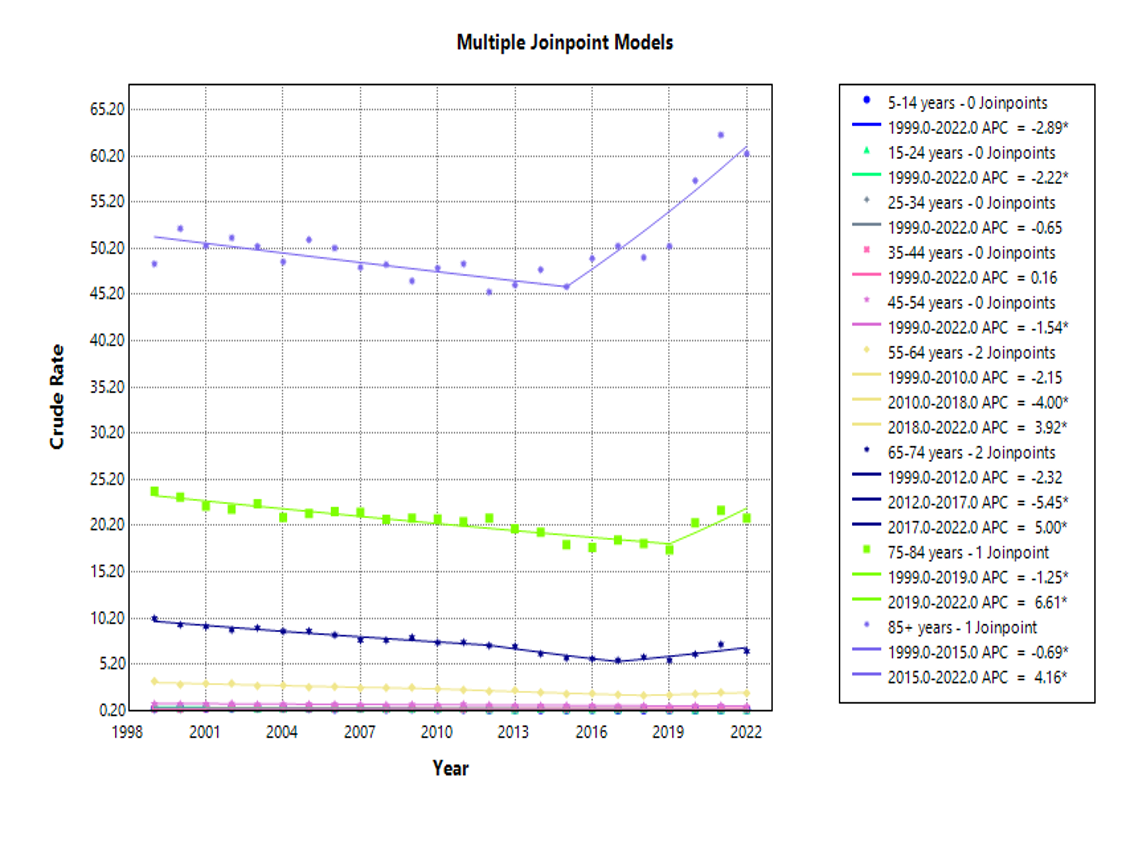


**Supplemental Figure 3**. Multiple Joint-Point Analysis stratified by age in the US, 1999-2022

| **Year** | **5-14** | **15-24** | **25-34** | **35-44** | **45-54** | **55-64** | **65-74** | **75-84** | **85+** |
| --- | --- | --- | --- | --- | --- | --- | --- | --- | --- |
| 1999 | 0.38 | 0.54 | 0.38 | 0.37 | 0.92 | 3.35 | 10.15 | 23.93 | 48.53 |
| 2000 | 0.46 | 0.51 | 0.29 | 0.35 | 0.89 | 2.98 | 9.46 | 23.3 | 52.36 |
| 2001 | 0.43 | 0.57 | 0.40 | 0.40 | 0.91 | 3.11 | 9.26 | 22.31 | 50.50 |
| 2002 | 0.46 | 0.55 | 0.36 | 0.46 | 0.95 | 3.12 | 8.9 | 21.95 | 51.36 |
| 2003 | 0.47 | 0.48 | 0.35 | 0.43 | 0.85 | 2.84 | 9.12 | 22.57 | 50.42 |
| 2004 | 0.43 | 0.46 | 0.34 | 0.39 | 0.87 | 2.91 | 8.75 | 21.07 | 48.75 |
| 2005 | 0.39 | 0.51 | 0.37 | 0.39 | 0.88 | 2.7 | 8.79 | 21.52 | 51.16 |
| 2006 | 0.35 | 0.51 | 0.38 | 0.39 | 0.88 | 2.76 | 8.36 | 21.73 | 50.25 |
| 2007 | 0.46 | 0.47 | 0.37 | 0.41 | 0.81 | 2.61 | 7.79 | 21.65 | 48.16 |
| 2008 | 0.35 | 0.43 | 0.38 | 0.37 | 0.78 | 2.64 | 7.79 | 20.86 | 48.46 |
| 2009 | 0.37 | 0.40 | 0.44 | 0.4 | 0.84 | 2.67 | 8.09 | 20.99 | 46.69 |
| 2010 | 0.35 | 0.40 | 0.38 | 0.41 | 0.78 | 2.53 | 7.53 | 20.91 | 48.06 |
| 2011 | 0.34 | 0.41 | 0.32 | 0.35 | 0.87 | 2.41 | 7.56 | 20.6 | 48.53 |
| 2012 | 0.29 | 0.37 | 0.31 | 0.39 | 0.78 | 2.26 | 7.21 | 21.02 | 45.49 |
| 2013 | 0.3 | 0.32 | 0.35 | 0.42 | 0.74 | 2.38 | 7.13 | 19.87 | 46.25 |
| 2014 | 0.29 | 0.40 | 0.34 | 0.43 | 0.74 | 2.14 | 6.32 | 19.49 | 47.9 |
| 2015 | 0.28 | 0.39 | 0.38 | 0.37 | 0.74 | 1.97 | 5.85 | 18.14 | 46.06 |
| 2016 | 0.31 | 0.37 | 0.37 | 0.44 | 0.71 | 2.03 | 5.79 | 17.83 | 49.14 |
| 2017 | 0.24 | 0.38 | 0.30 | 0.41 | 0.65 | 1.89 | 5.62 | 18.63 | 50.47 |
| 2018 | 0.25 | 0.41 | 0.30 | 0.41 | 0.69 | 1.81 | 5.96 | 18.25 | 49.22 |
| 2019 | 0.28 | 0.37 | 0.31 | 0.36 | 0.62 | 1.84 | 5.62 | 17.58 | 50.45 |
| 2020 | 0.24 | 0.32 | 0.29 | 0.45 | 0.71 | 1.98 | 6.27 | 20.48 | 57.54 |
| 2021 | 0.27 | 0.32 | 0.34 | 0.42 | 0.75 | 2.13 | 7.34 | 21.84 | 62.5 |
| 2022 | 0.29 | 0.34 | 0.33 | 0.38 | 0.61 | 2.06 | 6.59 | 20.98 | 60.47 |

**Supplemental Table 3**. Average crude mortality by age in the US 1999-2022.


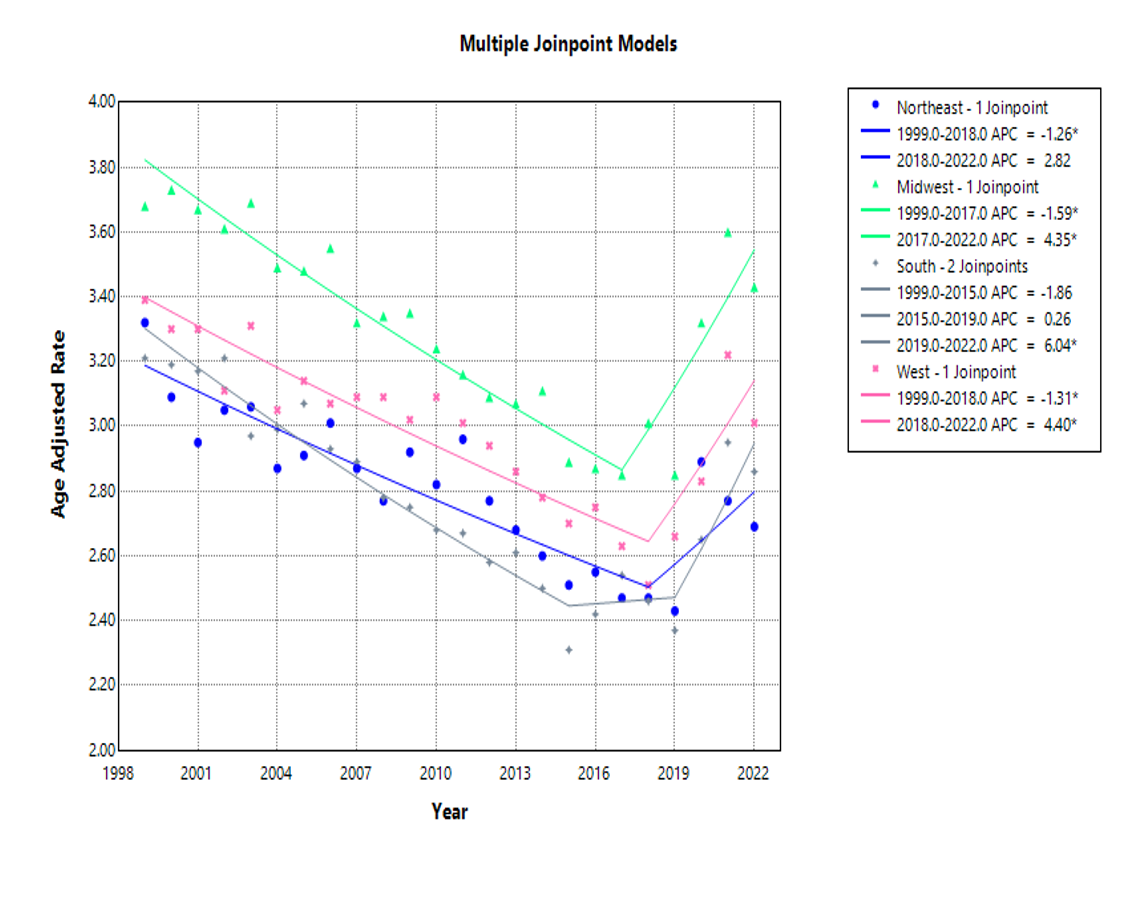
 **Supplemental Figure 4**. Multiple Joint-Point Analysis stratified by region in the US, 1999-2022

| **Year** | **Northeast** | **Midwest** | **South** | **West** |
| --- | --- | --- | --- | --- |
| 1999 | 3.32 | 3.68 | 3.21 | 3.39 |
| 2000 | 3.09 | 3.73 | 3.19 | 3.30 |
| 2001 | 2.95 | 3.67 | 3.17 | 3.30 |
| 2002 | 3.05 | 3.61 | 3.21 | 3.11 |
| 2003 | 3.06 | 3.69 | 2.97 | 3.31 |
| 2004 | 2.87 | 3.49 | 2.99 | 3.05 |
| 2005 | 2.91 | 3.48 | 3.07 | 3.14 |
| 2006 | 3.01 | 3.55 | 2.93 | 3.07 |
| 2007 | 2.87 | 3.32 | 2.89 | 3.09 |
| 2008 | 2.77 | 3.34 | 2.78 | 3.09 |
| 2009 | 2.92 | 3.35 | 2.75 | 3.02 |
| 2010 | 2.82 | 3.24 | 2.68 | 3.09 |
| 2011 | 2.96 | 3.16 | 2.67 | 3.01 |
| 2012 | 2.77 | 3.09 | 2.58 | 2.94 |
| 2013 | 2.68 | 3.07 | 2.61 | 2.86 |
| 2014 | 2.60 | 3.11 | 2.50 | 2.78 |
| 2015 | 2.51 | 2.89 | 2.31 | 2.70 |
| 2016 | 2.55 | 2.87 | 2.42 | 2.75 |
| 2017 | 2.47 | 2.85 | 2.54 | 2.63 |
| 2018 | 2.47 | 3.01 | 2.46 | 2.51 |
| 2019 | 2.43 | 2.85 | 2.37 | 2.66 |
| 2020 | 2.89 | 3.32 | 2.65 | 2.83 |
| 2021 | 2.77 | 3.6 | 2.95 | 3.22 |
| 2022 | 2.69 | 3.43 | 2.86 | 3.01 |

**Supplemental Table 4**. Age-adjusted morality by age in the US from 1999-2022.


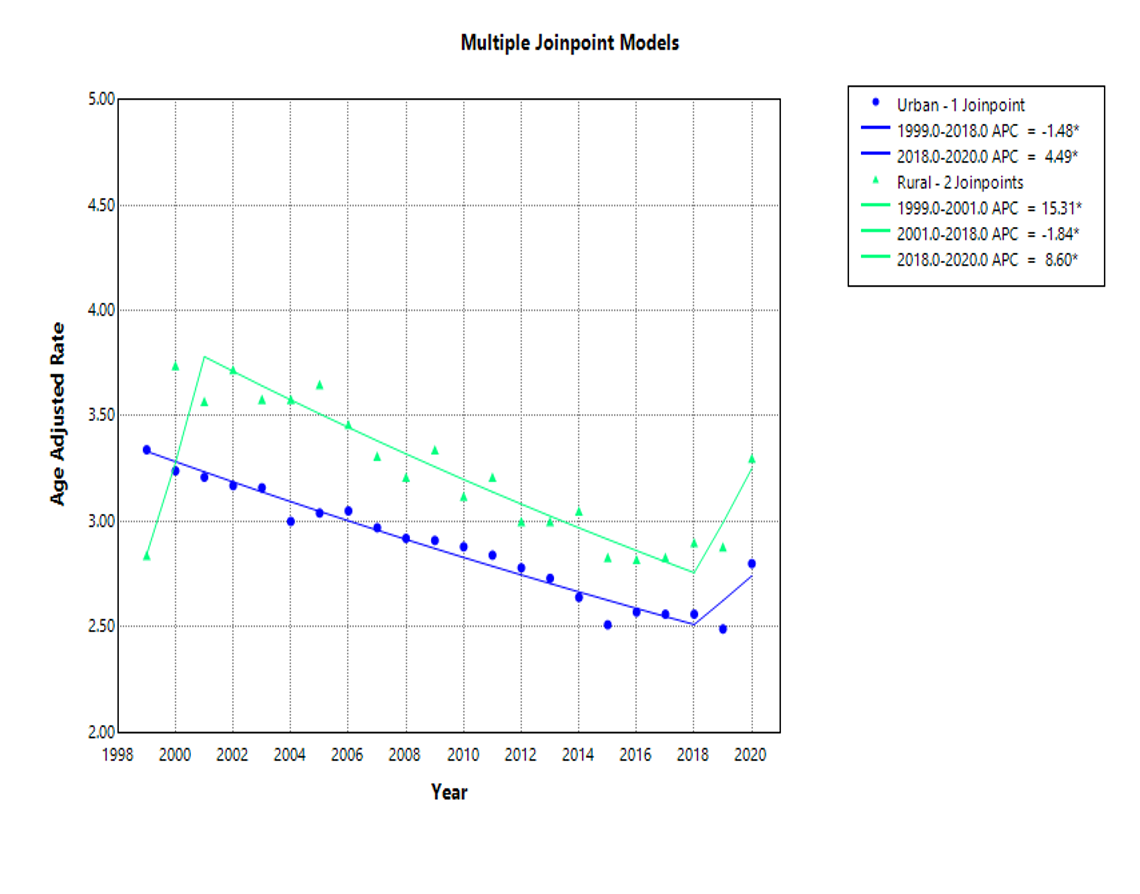


**Supplemental Figure 5**. Multiple Joint-Point Analysis stratified by rural vs. urban in the US, 1999-2020.

| **Year** | **Urban** | **Rural** |
| --- | --- | --- |
| 1999 | 3.34 | 3.64 |
| 2000 | 3.24 | 3.74 |
| 2001 | 3.21 | 3.57 |
| 2002 | 3.17 | 3.72 |
| 2003 | 3.16 | 3.58 |
| 2004 | 3.00 | 3.58 |
| 2005 | 3.04 | 3.65 |
| 2006 | 3.05 | 3.46 |
| 2007 | 2.97 | 3.31 |
| 2008 | 2.92 | 3.21 |
| 2009 | 2.91 | 3.34 |
| 2010 | 2.88 | 3.12 |
| 2011 | 2.84 | 3.21 |
| 2012 | 2.78 | 3.00 |
| 2013 | 2.73 | 3.00 |
| 2014 | 2.64 | 3.05 |
| 2015 | 2.51 | 2.83 |
| 2016 | 2.57 | 2.82 |
| 2017 | 2.56 | 2.83 |
| 2018 | 2.56 | 2.90 |
| 2019 | 2.49 | 2.88 |
| 2020 | 2.80 | 3.30 |

**Supplemental Table 5**. Age-Adjusted Mortality rural vs. urban from in the US from 1999-2020.
